# Grammatical Comprehension in Italian Children with Autism Spectrum Disorder

**DOI:** 10.3390/brainsci10080510

**Published:** 2020-08-02

**Authors:** Jessica Barsotti, Gloria Mangani, Roberta Nencioli, Lucia Pfanner, Raffaella Tancredi, Angela Cosenza, Gianluca Sesso, Antonio Narzisi, Filippo Muratori, Paola Cipriani, Anna Maria Chilosi

**Affiliations:** 1 IRCCS Stella Maris Foundation, Calambrone, 56018 Pisa, Italy; jessica.barsotti@fsm.unipi.it (J.B.); gloria.man96@hotmail.it (G.M.); roberta.nencioli@fsm.unipi.it (R.N.); lucia.pfanner@fsm.unipi.it (L.P.); raffaella.tancredi@fsm.unipi.it (R.T.); angela.cosenza@fsm.unipi.it (A.C.); gianluca.sesso@fsm.unipi.it (G.S.); antonio.narzisi@fsm.unipi.it (A.N.); paola.cipriani@fsm.unipi.it (P.C.); anna.chilosi@fsm.unipi.it (A.M.C.); 2 Department of Clinical and Experimental Medicine, University of Pisa, Via Savi, 10, 56126 Pisa, Italy

**Keywords:** autism spectrum disorder, language profiles, grammatical comprehension

## Abstract

Language deficits represent one of the most relevant factors that determine the clinical phenotype of children with autism spectrum disorder (ASD). The main aim of the research was to study the grammatical comprehension of children with ASD. A sample of 70 well-diagnosed children (60 boys and 10 girls; aged 4.9–8 years) were prospectively recruited. The results showed that language comprehension is the most impaired language domain in ASD. These findings have important clinical implications, since the persistence of grammatical receptive deficits may have a negative impact on social, adaptive and learning achievements. As for the grammatical profiles, persistent difficulties were found during the school-age years in morphological and syntactic decoding in children with relatively preserved cognitive and expressive language skills. These data and the lack of a statistically significant correlation between the severity of ASD symptoms and language skills are in line with the DSM-5 (Diagnostic and Statistical Manual of Mental Disorders, Fifth Edition) perspective that considers the socio-communication disorder as a nuclear feature of ASD and the language disorder as a specifier of the diagnosis and not as a secondary symptom anymore. The presence of receptive difficulties in school-age ASD children with relatively preserved non-verbal cognitive abilities provides important hints to establish rehabilitative treatments.

## 1. Introduction

Children with autism spectrum disorder (ASD) show heterogeneous functional profiles and outcomes [1,2]. Language deficits represent one of the most relevant factors that determine the clinical phenotype of children with ASD. Language deficits in ASD children can be described as a continuum, as on one hand there are non-verbal or minimally verbal children or those who do not acquire verbal language (in variable percentages up to 50% according to different studies) [3,4,5,6,7,8], and on the other hand there are children with formally appropriate but pragmatically inadequate language [9,10]. Within this continuum, highly variable language profiles can be found, with children exhibiting language delay, children with language disorders, and those who, despite reaching their first language milestones (i.e., speaking their first words between 12 and 18 months), later experience an arrest or a regression in language development. 

Though communication and language in children with ASD have been extensively investigated [4,9,10,11,12,13], the nature of the language deficit still remains an open issue. According to the DSM-5 [14], social communication disorder is considered a nuclear feature of ASD, while language disorder is only defined as a “specifier” of the condition. Some authors consider language disorders and ASD as potentially comorbid conditions [10,15] and use the term ASD-LI to refer to individuals with ASD who have impairments in structural language, regardless of their overall cognitive functioning.

The majority of published studies report that, in ASD, receptive language is more impaired than expressive language [5,11,16,17,18,19,20,21], but this finding has not been completely confirmed [22,23]. Moreover, the issue of the discrepancy between receptive and expressive competences is still unclear in terms of whether it should be considered as a possible marker of ASD, or whether both receptive and expressive language difficulties are comorbid to ASD.

Receptive language disorders in children with ASD are part of a wide and more complex phenotype characterized by pragmatic and neuropsychological deficits that may impair the acquisition of verbal comprehension. According to Tager-Flusberg and colleagues [24], the ASD’s problems of language comprehension are especially present in everyday situations, rather than during single-word comprehension testing. Children with ASD exhibit impairments in the ability to decode relevant contextual cues and deficits in social attention [25,26,27,28], whereas typically developing (TD) children are able from an early age to identify and select salient sensory stimuli and crucial social cues that are relevant both for comprehension and communication [29,30].

Grammatical comprehension, as a specific language skill that is required for decoding verbal messages in interactions, would represent a crucial matter of research in the field of ASD. However, it is worth noticing that relatively few studies are available which assess the comprehension of someone with ASD by means of specific tasks that differentially evaluate their lexical and grammatical skills. The evaluation of receptive abilities in children with ASD is of crucial importance in the clinical context in order to more clearly define the language profile, which has always been considered a paramount prognostic marker of development. Indeed, minimally verbal children with ASD exhibit a greater severity of autistic symptoms and globally worse clinical outcomes [31] than those with normal or mildly delayed language development, for whom an outcome could be more satisfactory [32]. Considering the above mentioned prognostic significance of language skills on the developmental perspective of children with ASD, the aims of the present study were:

1. To assess grammatical receptive skills in relation to other language abilities;
2. To investigate the relative contributions of non-verbal cognitive abilities and the severity of autistic symptoms on the language profile of children with ASD;
3. To examine the qualitative and quantitative differences in grammatical comprehension between ASD and TD children.

## 2. Materials and Methods

### 2.1. Sample

The present study was conducted on a sample of 70 children (60 boys) aged 4.9–8 years (mean age: 6.3 years; SD: 11 months; age range: 4.9–8 years) prospectively recruited at IRCCS Stella Maris Foundation (Calambrone, Pisa, Italy) from February 2009 to May 2018.

Inclusion criteria were as follows:

- A diagnosis of either autistic disorder according to DSM-IV-TR criteria or autism spectrum disorder according to DSM-5 criteria;
- Average or borderline non-verbal intellectual or developmental functioning level assessed through standardized psychometric tests;
- Expressive language at the level of multiword productions.

In order to qualitatively analyze grammatical comprehension, a subsample of 54 children with ASD and performance IQ ≥ 85 was selected and compared to 54 age- and sex-matched typically developing (TD) children. TD children were recruited from kindergarten and elementary schools in the area of Pisa, excluding subjects exposed to bilingualism.

Both groups were subdivided into four age groups at 12 months intervals, from 4.10 to 8 years. Informed written consent was obtained from the parents of all participants. This study was approved by the Pediatric Ethical Committee of the Tuscany Region (approval number: 178/2016) and was conducted according to the Helsinki Declaration.

### 2.2. Tests and Procedures

Grammatical comprehension was assessed using the Grammatical Comprehension Test for Children (Test Comprensione Grammaticale per Bambini; TCGB) [33] standardized on Italian children aged 3.6–8 years. The TCGB is a picture multiple-choice language test composed of 76 sentences pertaining to eight main blocks of grammatical structures: locatives, inflectionals, both affirmative and negative actives and passives, relatives and datives. Within each block of structures, the clauses differ not only in grammatical complexity, but also in semantic complexity (i.e., irreversible vs. reversible and probable vs. improbable clauses).

Children with ASD also underwent a comprehensive assessment of language skills by means of a receptive vocabulary test (PPVT-R: Peabody Picture Vocabulary Test—Revised) [34], an expressive picture naming test (One-Word Picture Vocabulary Test) [35] and the analysis of spontaneous language performed according to a six level rating system (Grid of Analysis of Spontaneous speech—GASS) [36,37]. For a detailed description of the TCGB and GASS, see Tables S1 and S2 in the Supplementary Materials. For all language tests, *z*-scores below—1.5 SD of the mean were considered as deficient.

*Wechsler Preschool and Primary Scale of Intelligence*, 3rd Ed (WPPSI-III, [38]) performance IQ, the Perceptual Reasoning Index at WISC-IV [39] or Griffiths [40] developmental quotient of the performance scale were used as measures of the non-verbal intellectual functioning level.

Finally, the Autism Diagnostic Observation Schedule-Generic (ADOS-G) [41] and Autism Diagnostic Observation Schedule-Second Edition (ADOS-2) [42] semi-structured observations were performed in ASD children for the evaluation of the autistic symptomatology severity.

### 2.3. Statistical Analysis

Skewness and Kurtosis statistics did not demonstrate a normal distribution for language-related variables, thus non-parametric tests were utilized. On the whole sample of 70 children with ASD, Spearman’s rank correlation coefficients between the grammatical comprehension non-verbal cognitive scores and ADOS severity scores were calculated. The Mann–Whitney U test was used to compare: (1) the non-verbal cognitive scores between children who performed averagely in the TCGB and children whose performances were classed as deficient; (2) the TCGB scores between the 54 ASD and the 54 TD children. The Kruskal–Wallis test for independent samples was also used to assess significant differences between the four age groups (5, 6, 7 and 8-year-old groups) of 54 children with ASD and 54 TD controls. Statistical analyses were performed using SPSS 21 software (IBM SPSS Statistics, Chicago, IL, USA).

## 3. Results

### 3.1. Language Profiles

Considering the mean z scores of the whole sample (see Table 1), grammatical comprehension appeared the most impaired domain compared to the other language measures. The TGCB mean total z score and the mean z score of the different structures (with the exception of active negative sentences) fell below −1.5 SD of the mean. Sixty-three percent of children with ASD had impaired grammatical comprehension. As for the vocabulary measures, the mean receptive lexical quotient at the PPVT was in the borderline range, with 56% of children showing an impaired performance. The expressive vocabulary scores were in the average range both for high and low frequency words (only 18% of children exhibited an impaired performance). According to the GASS, expressive language was at level 4, which corresponds to a deficient control of complex grammar, and only 14% of children had a more severe deficit.

### 3.2. Correlations between Language Measures, Non-Verbal Cognitive Skills and Autistic Symptoms Severity

Statistically significant correlations were found between the non-verbal IQ and total TCGB scores (*p* = 0.020). Non-verbal IQ was also correlated with active negative and passive negative clauses (*p* = 0.000 and *p* = 0.023, respectively). However, it is worth noting that non-verbal cognitive abilities did not differ significantly between ASD children with average and impaired comprehensions (*p* = 0.073).

The severity of the autistic symptoms assessed through the ADOS was low/moderate and did not correlate significantly with any expressive and receptive language measure.

### 3.3. Grammatical Comprehension Profiles of ASD and TD Children

The subsample of 54 children with ASD with a non-verbal IQ ≥ 85, was subdivided into four age groups (i.e., 5, 6, 7 and 8-year-old groups). These subgroups did not differ significantly in terms of non-verbal cognitive abilities (*p* = 0.296) or the severity of autistic symptoms (*p* = 0.212).

**Figure 1:**
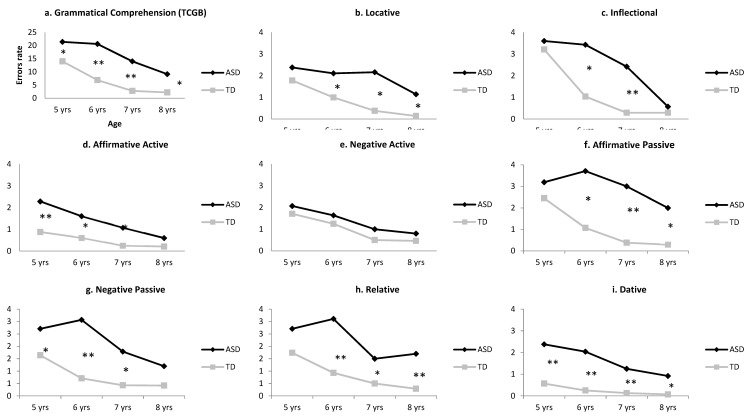
ASD and TD children’s total (**a**) and single clause type (**b**–**i**) error scores (* *p* ≤ 0.05; ** *p* ≤ 0.001). Abbreviation: yrs: years.

As displayed in Table 2 and Figure 1, the comparison between ASD and TD children showed that ASD children had significantly lower scores than TD children for most types of grammatical clauses at different ages. As expected, performances of both ASD and TD children changed with age; however, the ASD children’s performances changed at a significantly slower rate compared to the TD children. In the 5-year-old subgroup, statistically significant differences emerged for affirmative active, negative passive and dative clauses, whereas, in the 6- and 7-year-old subgroup, ASD children scored significantly lower than TD children in all types of clauses (with the exception of negative active clauses). The 8-year-old subgroup showed a significant reduction in their error scores, compared to 5- and 6-year-old children (*p* = 0.030 and *p* = 0.010, respectively). Nonetheless, significant differences between ASD and TD children were still evident for locative, affirmative passive, relative and dative clauses. Considering the semantic and grammatical complexity of the different clause types (for details see Table 3), markedly significant differences between ASD and TD children were observed for the more complex but not for the simpler sentences (i.e., verbal vs. nominal inflexions, projective vs. topological locatives, semantically improbable vs. probable and neutral active affirmative clauses, reversible vs. irreversible clauses) [33]. The comparison between the reversible and irreversible clauses total scores showed a highly significant difference between TD and ASD children in the comprehension of reversible clauses (*p* = 0.000), but not in irreversible clauses (*p* = 0.834). 

## 4. Discussion

The present research is the first Italian study aimed at assessing grammatical comprehension in children with ASD. Our results support the hypothesis proposed by some authors [9,11,17,20,43] that language comprehension would be the most impaired language domain in autism, in spite of its highly heterogeneous linguistic phenotype [1]. Around two thirds of our patients exhibited an impaired grammatical comprehension, whereas grammatical production was impaired in only 14% of children. Additionally, in the lexical domain, receptive skills were more impaired than expressive ones. However, the deficit of language comprehension was not homogeneous, since receptive grammar was more impaired than receptive vocabulary. The above language profile differs from the one usually observed in children with a primary developmental language disorder, which is generally characterized by better receptive abilities as opposed to expressive language abilities [16,44,45,46]. The discrepancy between receptive and expressive skills in children with ASD is, however, still a debated issue, because it is not clear whether it could be considered a specific marker of ASD.

As for the relationship between language and non-verbal cognitive abilities, a positive correlation between non-verbal intellectual functioning and grammatical comprehension was found, but non-verbal cognitive abilities did not differ statistically between children with a normal comprehension and those with an impaired comprehension. These apparently conflicting findings suggest that the grammar comprehension deficit is quite specific in children with ASD, and, thus, is relatively independent from non-verbal cognitive skills. This evidence is in line with the results of a study conducted on a sample of ASD children with average or borderline intellectual functioning [43] in which non-verbal IQ explained a limited amount of the variance of language scores.

The lack of statistically significant correlations between the severity of ASD symptoms and language skills seems to confirm the DSM-5’s choice to let language deficits outside the nuclear symptoms of autism. In this way, the DSM-5 avoids the risk of automatically establishing an overlap between communication and structural language skills.

### 4.1. Grammatical Comprehension Profiles in Children with ASD and TD

To date, the qualitative assessment of grammatical decoding strategies in children with ASD has generally been carried out on English-speaking subjects [47,48,49], with few reports in the recent literature [50]. In the present Italian study, the qualitative assessment of language comprehension showed significant differences between TD and ASD children. The latter showed both delayed and atypical receptive grammatical skills.

Data on the development of grammatical comprehension in TD children show a significant change between 5 and 6 years of age, a period characterized by an increasing integration of the different decoding strategies, and by a sharp reduction in the inter-individual variability. By 7 or 8 years old, TD children show a further increase in their capacity to generalize the use of linguistic principles and rules for decoding more complex grammatical structures [33]. In children with ASD, improvements in grammatical comprehension occurred around 7 or 8 years of age, but their receptive skills remained significantly impaired in comparison to TD children. This result has important clinical implications, since the persistence of grammatical receptive deficits may have a negative impact on social, adaptive and learning achievements during the school-age years [43,51,52].

As for the grammatical profiles, persistent difficulties were found in morphological (i.e., inflectional clauses) and syntactic decoding (i.e., locative, passive, relative and dative clauses). Similar to what was observed in TD children, the comprehension of active negative sentences was more difficult than the comprehension of active affirmative sentences. This might be due to the poor representability of negative actions and to the fact that decoding of such structures requires inferential reasoning (e.g., the correct answer for the sentence “the child does not eat the soup” is represented by a picture in which the child is eating an ice-cream).

Relative, passive and dative clauses were more difficult for children with ASD than for TD children. In the case of relative clauses, the persistence of difficulties up to 8 years of age probably depends on their length and structural complexity. For passive and dative clauses, the crucial factor may be the reversibility of actions. The significant difficulty in decoding reversible, but not irreversible sentences, which characterizes the profiles of children with ASD, is an important result because it suggests the presence of a specific linguistic deficit. In fact, in reversible sentences the roles of the agent or patient of the action are interchangeable, as they are both animate referents (e.g., “the girl pushes the boy” or vice versa). Therefore, decoding these sentences is crucially based on the word order, that is, on syntactic strategies. Conversely, in irreversible sentences, the agent and patient roles are not interchangeable, because of semantic restrictions linked to the animate/inanimate nature of referents (e.g., “the car is washed by the boy”). In this case, lexical factors can drive the sentence decoding. Difficulties in processing reversible sentences have also been described [47] in a study reporting that impairments in reversible sentence comprehension was greater than expected from the global level of receptive skills. We would suggest that ASD children with average or borderline intellectual functioning may be able to process lexical information, while showing difficulties to acquire an integrate system for decoding morpho-syntactic information.

### 4.2. Limitations

This study presents several limitations. A first limitation is that the non-verbal IQ tests we chose to administer require verbal comprehension skills to follow the instructions, which might have contributed to the positive correlation between the non-verbal IQ scores and the grammatical comprehension scores. Another limit is the typology of our structured picture-based comprehension test, which does not tap into ecologically natural and contextual-based comprehension abilities, thus not providing a comprehensive profile of ASD children’s receptive skills.

Finally, although there was no a priori restriction on patient selection according to the severity of autistic symptomatology applied in the present study, the children with ASD showed a low to moderate severity of autistic symptomatology. This may be due to the fact that only patients who were able to complete the structured language evaluation were included, creating a bias in the selection of the sample. Patients on the autism spectrum exhibit quite heterogeneous clinical pictures, while the present study focuses on the language comprehension of intellectually unimpaired verbal children with a low/moderate severity of autistic symptoms. Hence, our results may not be generalizable to the whole population of patients with ASD.

## 5. Conclusions

Our results are in line with the DSM-5 perspective according which emphasizes that the socio-communication disorder is a nuclear feature of ASD, whereas the language disorder should be considered as a specifier of the diagnosis, not a nuclear deficit or a secondary symptom. Among the language abilities we have described, a specific receptive grammatical deficit was found, whilst cognitive and expressive language are relatively preserved. It would be important in the future to evaluate the relationship between language comprehension and some neuropsychological abilities, such as working memory and executive functions to shed some light on the nature of the comprehension deficits in children with ASD.

The presence of receptive difficulties in school-age ASD children with relatively preserved non-verbal cognitive abilities provides important hints about treatment. Receptive language difficulties, when not associated with expressive deficits, may go unnoticed [43] and may be neglected despite their relevance in everyday life functioning, communication and social interaction. Early support to aid in the acquisition of adequate receptive language skills is crucial for further development. Moreover, as grammatical comprehension difficulties tend to persist in ASD children up to school-age (when generally speech therapy gradually diminishes), a specific intervention on both oral and written receptive skills is mandatory.

## Figures and Tables

**Table 1:** Sample characteristics (*n* = 70).

Children’s Language, Cognitive and ADS Profiles	Mean	SD	Mean z Score
**Age**	76.43	11.80	-
**Grammatical Comprehension (TCGB)**	20.33	11.27	−3.25
Locative	2.55	2.14	−1.66
Inflectional	3.29	2.47	−2.55
Affirmative Active	1.99	1.98	−2.59
Negative Active	1.90	1.68	−0.86
Affirmative Passive	3.31	2.29	−2.58
Negative Passive	2.74	1.79	−2.61
Relative	2.59	1.85	−2.97
Dative	1.96	1.33	−4.73
**Receptive Vocabulary (QL PPVT-R)**	80.72	11.12	−1.22
**Grammatical production level (GASS)**	4.18	0.62	-
**Expressive Vocabulary for high-frequency words (Brizzolara)**	13.58	5.27	−0.38
**Expressive Vocabulary for low-frequency words (Brizzolara)**	32.32	6.09	−0.77
**NVIQ**	100.62	14.66	-
**ADOS Comparison Score**	5.30	1.42	-

Abbreviations: SD: standard deviation; NVIQ: non-verbal intelligence quotient.

**Table 2:** Comparison between typically developing (TD) and autism spectrum disorder (ASD) children in the different age ranges.

Grammatical Comprehension	5 Years			6 Years			7 Years			8 Years		
	TD (*n* = 21)	ASD (*n* = 21)	*p*	TD (*n* = 14)	ASD (*n* = 14)	*p*	TD (*n* = 12)	ASD (*n* = 12)	*p*	TD (*n* = 7)	ASD (*n* = 7)	*p*
TCGB Total score	14.00 (6.51)	21.38 (8.95)	0.004	6.86 (2.64)	20.68 (3.27)	0.000	2.79 (1.12)	14.00 (7.48)	0.000	2.21 (2.02)	9.14 (4.92)	0.005
Locative	1.79 (1.52)	2.38 (1.84)	ns	1.00 (0.70)	2.11 (1.36)	0.012	0.38 (0.57)	2.17 (1.70)	0.002	0.14 (0.24)	1.14 (0.85)	0.011
Inflectional	3.21 (2.22)	3.60 (2.40)	ns	1.04 (1.15)	2.33 (0.62)	0.002	0.29 (0.33)	2.42 (1.04)	0.000	0.29 (0.39)	0.57 (0.45)	ns
Affirmative Active	0.88 (0.74)	2.29 (1.55)	0.001	0.61 (0.56)	1.61 (1.66)	0.043	0.25 (0.50)	1.08 (1.28)	0.047	0.21 (0.57)	0.64 (0.80)	ns
Negative Active	1.71 (1.27)	2.07 (1.54)	ns	1.25 (1.03)	1.64 (1.26)	ns	0.46 (0.58)	1.00 (1.19)	ns	0.50 (0.58)	0.79 (0.99)	ns
Affirmative Pass.	2.45 (2.64)	3.19 (2.11)	ns	1.07 (1.47)	3.71 (2.55)	0.003	0.38 (0.38)	3.00 (2.13)	0.000	0.29 (0.57)	2.00 (1.68)	0.025
Negative Passive	1.64 (1.43)	2.71 (1.51)	0.023	0.71 (0.80)	3.07 (1.20)	0.000	0.42 (0.51)	1.79 (1.47)	0.006	0.43 (0.53)	1.21 (1.55)	ns
Relative	1.74 (1.34)	2.74 (1.86)	ns	0.93 (0.92)	3.11 (1.91)	0.001	0.50 (0.56)	1.50 (1.46)	0.038	0.29 (0.39)	1.71 (0.81)	0.001
Dative	0.57 (0.81)	2.38 (1.22)	0.000	0.25 (0.38)	2.04 (1.42)	0.000	0.13 (0.31)	0.92 (0.63)	0.001	0.07 (0.19)	1.07 (0.67)	0.003

**Table 3:** Comparison between ASD and TD children: qualitative assessment of the clause type related subcategories.

Structure	Sub-Categories	TD (*n* = 54)	ASD (*n* = 54)	Mann–Whitney Test
Locative	topological	0.12 (0.34)	0.42 (0.88)	0.021
	projective	0.94 (1.07)	1.57 (1.31)	0.009
Inflectional	nominal	0.07 (0.20)	0.16 (0.50)	ns
	verbal	0.84 (1.14)	1.80 (1.46)	0.000
	possessive	0.70 (1.16)	0.89 (0.92)	0.027
Affirmative Active	SV (reflexive)	0.08 (0.30)	0.41 (0.46)	0.000
	reversible probable	0.11 (0.21)	0.05 (0.18)	0.048
	reversible neutral	0.13 (0.28)	0.28 (0.50)	ns
	reversible improbable	0.07 (0.26)	0.54 (0.66)	0.000
	reversible with subject object inanimate-animate	0.19 (0.37)	0.30 (0.65)	ns
Negative Active	SV	0.01 (0.07)	0.13 (0.48)	0.041
	SVO irreversible	0.50 (0.67)	0.43 (0.65)	ns
	SVO reversible	0.65 (0.84)	1.00 (0.92)	0.023
Affirmative Passive	irreversible	0.53 (1.08)	0.40 (0.53)	ns
	reversible probable	0.22 (0.49)	0.94 (1.06)	0.000
	reversible improbable	0.35 (0.55)	0.95 (0.84)	0.000
	reversible neutral	0.25 (0.59)	0.78 (1.02)	0.000
Negative Passive	SV	0.10 (0.36)	0.77 (0.72)	0.000
	SVA irreversible	0.34 (0.53)	0.55 (0.68)	ns
	SVA reversible	0.53 (0.88)	1.05 (0.96)	0.002
Relative	embedded	0.61 (0.70)	1.44 (1.09)	0.000
	right branching	0.45 (0.75)	0.92 (1.12)	0.022
Dative	AAA	0.26 (0.56)	0.92 (0.85)	0.000
	AIA	0.07 (0.24)	0.86 (0.88)	0.000

Abbreviations: SV: subject verb; SVO: subject verb object; SVA: subject verb agent; AAA: animate animate animate; AIA: animate inanimate animate.

## Supplementary Materials

The following are available online at https://www.mdpi.com/2076-3425/10/8/510/s1, Table S1: The Test of grammatical Comprehension for Children TCGB, [33], Table S2: Grid of Analysis of Spontaneous Speech GASS.
