# Supplementary material for: Grammatical Comprehension in Italian Children with Autism Spectrum Disorder
Source: Brain Sci. 2020 Aug 2;10(8):510. doi: 10.3390/brainsci10080510 (PMC7464622; doi:10.3390/brainsci10080510)
Supplement: Supplementary file 1 [file brainsci-10-00510-s001.pdf]

Supplemental Material

Table 1. The Test of Grammatical Comprehension for Children TCGB [33].

Structures	Number of Items	Sub-Categories
Locative items	14	Topological (under/over; in/out; beside/far)<br>Projectives (front/back, from/to, between)
Inflectional items	16	Nominal (number → singolar/plural; gender→ male/female)<br>Verbal (number→ singolar/plural; tense→ present/past/future)<br>Possessive (singolar/plural)
Affirmative active items	10	SV: Subject Verb (i.e. "The mother is doing the washing")<br>Reversible probable (i.e. "The mother is combing the little girl's hair")<br>Reversible neutral (i.e. "The little boy is pushing the little girl")<br>Reversible improbable (i.e. "The little boy feeds the mother")<br>Reversible with subject object inanimate-animate (i.e. "The paper is burning the boy")
Negative active items	6	SV: Subject Verb (i.e. "The little boy isn't sleeping")<br>SVO irreversible: Subject Verb Object irreversible (i.e. "The cat isn't eating the fish")<br>SVO reversible: Subject Verb Object reversible (i.e. "The little girl isn't pushing the boy")
Affermative passive items	10	Irreversible (i.e. "The apple is eaten by the little girl")<br>Reversible probable (i.e. "The girl is dressed by the mother")<br>Reversible improbable (i.e. "The dog is being bitten by the boy")<br>Reversible neutral (i.e. "The dog is being pulled by the man")
Negative passive items	6	SV (i.e. "The piano is not played")<br>SVA irreversible: Subject Verb Agent irreversible (i.e. "The apple isn't taken by the little girl")<br>SVA reversible: Subject Verb Agent reversible (i.e. "The boy is not pushed by the girl")
Relative items	8	Embedded (i.e. "The little boy who is on the table is eating the jam"),<br>Right Branching (i.e. "The father is holding the balloon that the little boy is bursting")
Dative items	6	AAA: Animate-animate-animate (i.e. "The swallow's taking the worm to the little bird")<br>AIA: Animate-inanimate-animate (i.e. "The father's taking the cigarettes to the little boy")

Abbreviations: SV: Subject Verb; SVO: Subject Verb Object; SVA: Subject Verb Agent; AAA: Animate Animate Animate; AIA: Animate Inanimate Animate.

Table 2. Grid of Analysis of Spontaneous Speech GASS.

GASS: Grid of Analysis of Spontaneous Speech				
**Level**	**Mean chronological age (months)**	**Age range (months)**	**Mean Length of Utterance (words)**	
Level 0	9	8-12	-	Pre-linguistic stage: spontaneous language production is limited to babbling, sounds, and sporadic single words.
Level 1	14	12-18	-	Holophrastic stage: true words are few in number but single word utterances begin to be used consistently.
Level 2	20	19-25	1.4	Early combinatorial or presyntactic stage: emergence of two and three word combinations but single word utterances prevail (about 80%).
Level 3	23	20-26	2.15	Telegraphic or protosyntactic stage: Emergence of subject-verb-object multiword structures that are ungrammatical for omission of free morphemes in most obligatory contexts
Level 4	27	24-31	2.6	Grammatical stage one: acquisition of full control of free morphology and basic rules of the main clause in simple sentences; most complex sentences still present omission of free morphemes and subordinate clause functors.
Level 5	31	28-34	3.1	Grammatical stage two: grammatical control extends to many types of complex sentences with production of well-formed, long and complex sentences.
